# The promising drugs included in WHO’s Solidarity Project: a choice based in scientific knowledge and institutional competencies

**DOI:** 10.1590/0074-02760200603

**Published:** 2021-09-01

**Authors:** Andréia Cristina Galina, Deise Sarzi, Larissa Campos de Medeiros, André Luiz Franco Sampaio, Jacqueline Leta

**Affiliations:** 1 Universidade Federal do Rio de Janeiro, Instituto de Bioquímica Médica Leopoldo de Meis, Programa de Educação, Gestão e Difusão em Biociências, Rio de Janeiro, RJ, Brasil; 2 Instituto Nacional de Tecnologia, Divisão de Comunicação, Rio de Janeiro, RJ, Brasil; 3 Fundação Oswaldo Cruz-Fiocruz, Instituto de Tecnologia em Fármacos-Farmanguinhos, Departamento de Farmacologia, Rio de Janeiro, RJ, Brasil

**Keywords:** Solidarity Program, COVID-19, epidemic, global science, scientific publications, bibliometrics

## Abstract

**BACKGROUND:**

In March 2020, the World Health Organization (WHO) launched the Solidarity Program, probably the largest global initiative to encourage and support research in four promising drugs, named Remdesivir, Hydroxychloroquine, β Interferon and the combination Lopinavir / Ritonavir, to reduce the mortality of Coronavirus disease 2019 (COVID-19).

**OBJECTIVES:**

Considering the potential impact of Solidarity Program to restrain the current pandemic, the present study aims to investigate whether it was designed upon indicators of scientific productivity, defined as the level of the production of new scientific knowledge and of the institutional capabilities, estimated in terms of scientific publications and technological agreements.

**METHODS:**

The scientific documents on Alphacoronavirus, Betacoronavirus, Gammacoronavirus and Coronavirus were retrieved from Scopus database while the technological agreements on coronavirus were obtained through Cortellis. As for the institutions and countries, we have considered the data on author’s affiliations in both set of data. For comparison, we included the analysis of documents related with other drugs or therapies, such as vaccines and antibodies, which were listed in a Clarivate’s report on coronaviruses research.

**FINDINGS:**

Most of the analysis refers to documents on Coronavirus, the largest group. The number of documents related to WHO’s drugs are almost five times higher than in the other groups. This subset of documents involves the largest and most diverse number of institutions and countries. As for agreements, we observed a smaller number of institutions involved in it, suggesting differences between countries in terms of technical and human capabilities to develop basic and/or clinical research on coronavirus and to develop new forms or products to treat or to prevent the disease.

**MAIN CONCLUSIONS:**

Hence, the results shown in this study illustrate that decisions taken by an international scientific body, as WHO, were mainly based in scientific knowledge and institutional competencies.

Although coronaviruses became popular in 2020, these types of viruses have been considered pathogens for several groups of animals, including humans, since decades. Today, it is widely known that coronaviruses are a large family of viruses with the potential to cause a spectrum of diseases ranging from a simple cold to a severe acute respiratory syndrome.^1^

In the 1960s, coronaviruses that infect humans were isolated for the first time from samples of nasal secretion of patients with symptoms of cold.^2^ Since then, seven types of human coronavirus (HCoV) have been identified, among which four seem to have a seasonal incidence, especially in countries with temperate climates,^3^ and are associated with mild symptoms of respiratory diseases (HCoV-229E, HCoV-OC43, HCoV-NL63 and HCoV-HKU1). Two other types include the acute respiratory syndrome virus (SARS-CoV), identified in 2002, and the Middle East syndrome virus (MERS-CoV), identified in 2012, both are associated with severe respiratory symptoms and high mortality rates.^3^ The last type, identified in 2019 and known as “new coronavirus” (SARS-CoV-2), is also associated with severe respiratory symptoms, but unlike previous ones, it is highly transmissible,^4^ ^,^ ^5^ which favored the rapid spreading of the current pandemic.

According to the National Centre for Biotechnology Information (NCBI/USA), coronaviruses belong to the family Coronaviridae, subfamily Orthocoronavirinae, which is divided into four genera: Alphacoronavirus, Betacoronavirus, Gammacoronavirus and Deltacoronavirus. Alpha and Betacoronavirus infect only mammals, while most gamma and Deltacoronavirus infect birds but it may also infect mammals.^6^ ^,^ ^7^ Out of the seven HCoV already identified, two belong to the genus Alphacoronavirus (HCov-229E and HCoV-NL63) and the other five belong to the genus Betacoronavirus.^7^

The Betacoronavirus SARS-CoV-2, that is, the new coronavirus is highly pathogenic and responsible for the coronavirus disease pandemic started in late 2019, named Coronavirus disease 2019 (COVID-19). This pandemic has been mobilising the global scientific community in the search for alternatives to diagnose and prevent new cases, as well as alternatives to treat patients already infected by SARS-CoV-2.^8^ ^,^ ^9^ ^,^ ^10^ The current efforts of scientists from different parts of the world to increase and to share rapidly the new knowledge on COVID or coronavirus have been discussed and presented in several studies.^11^ ^,^ ^12^ ^,^ ^13^ ^,^ ^14^ ^,^ ^15^

The growing literature about scientific publications on COVID or coronavirus at a time when the world still suffers from the pandemic has highlighted aspects including: the contribution of a specific country or region,^16^ ^,^ ^17^ comparison with the scientific publication on other viruses,^18^ publication analysis based in different information sources^19^ ^,^ ^20^ and about the faster editorial flow.^21^ Despite the wide thematic variety in this literature, we have not observed studies focusing on scientific publication on drugs and preventive treatments, such as vaccines, to restrain the new coronavirus.

The discovery and development of new drugs, as well as other treatments, can take years to result in a prototype and product.^10^ ^,^ ^22^ ^,^ ^23^ However, in an emergency, one way to fasten this process is the repositioning of drugs, that is, the investigation of a new use for an existing drug, thus avoiding expensive and time-consuming toxicological essays.^24^ ^,^ ^25^ Such initiatives have been taking place in the COVID-19 epidemic, being the Solidarity Program probably the largest example. Launched in March 2020 by the World Health Organization (WHO), the program aims encouraging and supporting worldwide research in four promising drugs:^26^ ^,^ ^27^ (i) remdesivir, an antiviral that inhibits the RNA virus cycle; (ii) hydroxychloroquine, a drug used as a therapy against malaria and rheumatoid arthritis; (iii) beta interferon, a drug used in the treatment of multiple sclerosis; and (iv) the combination lopinavir / ritonavir, drugs used to treat human immunodeficiency virus (HIV). After almost four months and in the face of negative results, WHO reviewed the program and withdrew the support for research with hydroxychloroquine and the combined drugs lopinavir/ritonavir, maintaining the program with the other two.

Although the motivation for choosing these drugs, and not others, is not clear in the Solidarity Project,^27^ it is expected that the WHO’s choice was based on existing scientific evidences and competencies that place them in a prominent position. However, we may not discard that national and even global initiatives, in this case related to the epidemic, can also be motivated by other aspects, such as politics. As for the current pandemic, many studies have investigated the endorsement of hydroxychloroquine by far-right leaders. Casarões & Magalhães,^28^ for instance, discuss such endorsement with a theoretical background based in the concept of medical populism, a common style incorporated by political authorities during a health emergency.

Considering both the lack of studies about research on drugs related to coronavirus and the potential impact of the Solidarity Project to restrain the current pandemic, the present study aims to investigate whether WHO’s project was designed upon indicators of scientific productivity, an axiom of Bibliometric, as proposed by Narin in 1976.^29^ Hence, we define scientific productivity as the already existing effort of the global research on coronavirus in terms of both the production of new scientific knowledge and the institutional capabilities. As for the institutional capabilities, we considered the most prolific institutions among documents on coronavirus and among agreements on coronavirus, a proxy to find out institutions with the capability of developing drugs or therapies against COVID-19.

Based in scientific documents and in agreements, we focused our analysis in two main aspects: (a) general trends of these documents, including the time trends of documents on each group of coronaviruses and the number of documents on Coronavirus group according to the type of research application and (b) the identification of both documents and agreements devoted to the drugs indicated in the Solidarity Project as well as documents devoted to other drugs and therapies, like vaccines selected in the report “Disease briefing: coronaviruses”^30^ and the main leading institutions and countries.

## MATERIALS AND METHODS

We used the controlled vocabulary Medical Subject Headings (MeSH) browser^31^ to select the terms that were used to elaborate the search strategy in Scopus. On April 25, 2020, by using the exact match term ‘coronavirus’ in the filters Full Word Search, Exact Match and Main Heading Terms, we found three main terms in MeSH Tree Structures, which we named *coronavirus groups*, as presented in Table I. Note that the Deltacoronavirus is not presented among the three main groups include in the MeSH Tree Structure since it did not appear as a branch of this tree but as a single term associated to coronavirus. Such absence explains its absence in the following analysis.

**TABLE I:** MeSH Tree Structure based in the exact match term *coronavirus*

Coronavirus		
Coronaviruses Deltacoronavirus Deltacoronaviruses Munia Coronavirus HKU13 Coronavirus HKU15 Coronavirus, Rabbit Rabbit Coronavirus Coronaviruses, Rabbit Rabbit Coronaviruses Bulbul Coronavirus HKU11 Thrush Coronavirus HKU12	Alphacoronavirus	Alphacoronavirus 1 Coronavirus 229E, Human​ Coronavirus NL63, Human Porcine Epidemic Diarrhea Virus
	Betacoronavirus	Betacoronavirus 1 Coronavirus, Rat Middle East Respiratory Syndrome Coronavirus Murine Hepatitis Virus SARS Virus
	Gammacoronavirus	Coronavirus, Turkey Infectious Bronchitis Virus

After testing variations of MeSH terms, we decided to collect data separately in four groups: a generalist and other three groups that correspond to coronavirus genera found in MeSH Tree. Below, we present the details of the search strategies used in Scopus that consist in a combination of terms and adapted terms found in MESH linked by “or” (a common Boolean operator), by an asterisk (a wildcard operator that replaces multiple characters anywhere in a word) or/and by a W/n (a proximity operator that indicates the n words are in between term 1 and term 2).

**Fig. 1:**
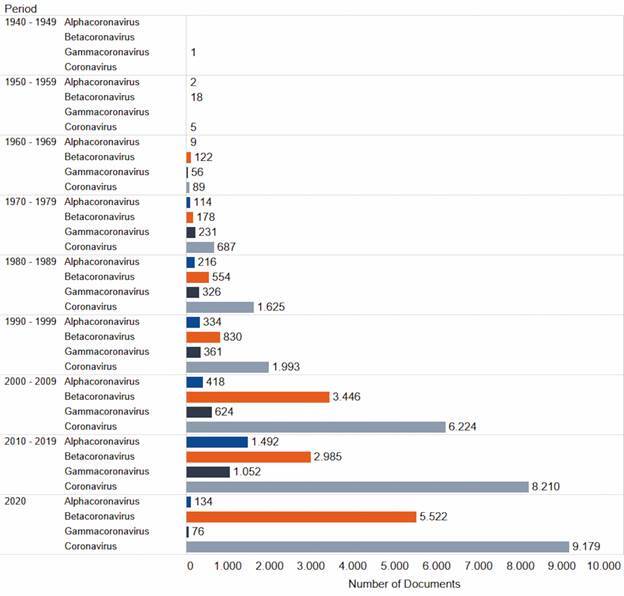
number of documents on coronavirus and on the three coronavirus families according to the decade. Source: Scopus.

- Coronavirus: coronav*;

- Group Alphacoronavirus: alphacoronav* OR “alpha coronav*” OR alphacoronavirus-1 OR “Transmissible gastroenteritis vir*” OR hcov-nl63 OR hcov-229e OR “Porcine epidemic diarrhea vir*”;

- Group Betacoronavirus betacoronavirus-1 OR betacoronav* OR beta-coronav* OR hcov-hku1 OR (“Porcine hemagglutinat*” W/1 “encephalomyelitis vir*”) OR “MERS vir*” OR mers-cov OR (murine W/1 “hepatitis vir*”) OR (mouse W/1 “Hepatitis Vir*”) OR (“Gastroenteritis Vir*” W/1 murine) OR mhv-jhm OR “SARS Vir*” OR (“Severe Acute Respiratory Syndrome” w/1 vir*) OR sars-cov*.

- Group Gammacoronavirus: gammacoronav* OR “Gamma coronav*” OR “Bluecomb Vir*” OR (“Transmissible Enteritis Vir*” W/2 turkey*) OR (“Enteri* Vir*” w/3 Turkey) OR (“bronchit* vir*” W/1 infect*);

Data collection was carried out on May 30, 2020 at the Scopus database. The choice of this database is justified by its larger journal collection when compared to other multidisciplinary databases and by the possibility of downloading 2,000 registers at once in different formats and with a diversity of metadata for each document. Using the advanced search mode, the search strategy of each group was searched in title, abstract and keyword filters. The results were exported in the BibTeX format, considering different metadata, as Bibliographical information and Abstract & keywords. The totals documents retrieved from Scopus in the four groups were: 2,719 on Alphacoronavirus, 13,655 on Betacoronavirus, 2,745 on Gammacoronavirus and 28,013 on Coronavirus. The later, that is, the most generalist and largest group, was used as reference for all analysis, except for Fig. 1.

The 28,013 documents on coronavirus were classified according to the type of research application as: vaccine, diagnosis and treatment. To identify documents classified as vaccine, we used the words *vaccine*, *vaccines*, *vaccinia* and *vaccination*. As for diagnosis, we used the *diagnos* while for those classified as treatment, we used the words *treat* and *therap*. For this process, we used a script developed in the R language^32^ and the words were searched in the title, abstract and database and author’s keywords of the 28,013 documents on coronavirus.

These documents on coronavirus were also classified according to the drug or therapy they are related with. For this purpose, we first identified documents related with WHO’s drugs, by searching in their title, abstract and keywords the following terms: “hydroxychloroquine sulfate”, remdesivir, “lopinavir / ritonavir”, “ritonavir / lopinavir”, “lopinavir plus ritonavir”, “ritonavir plus lopinavir”, “lopinavir ritonavir”, “ritonavir lopinavir”, “interferon beta”, “beta interferon”, “interferonbeta” and “betainterferon”.

As a comparative set of documents, we also identified documents related with other drugs or therapies (vaccines and antibodies) always considering the 28,013 documents on coronavirus. For this, we used the list of drugs and therapies listed in Clarivate’s report on coronaviruses research,^30^ the most comprehensive list of drugs and therapies related to coronaviruses by the time we started this study. Initially, we have selected 31 drugs or therapies all listed as clinical trials in phases I, II or III in Clarivate’s report. Nevertheless, we discarded 11 drugs and therapies since they were not found within documents on coronavirus. Thus, documents related to 20 drugs and therapies were classified in three groups: Other drugs, Antibody and Vaccine. The group Other drugs includes documents with at least one of the terms: “darunavir/cobicistat”, “cobicistat/darunavir”, “darunavir plus cobicistat”, cobicistat plus darunavir”, “darunavir cobicistat”, “cobicistat darunavir”, “chloroquine phosphate”, “danoprevir”, “diammonium glycyrrhiinate”, “favipiravir”, “leronlimab” or “oseltamivir phosphate”. As for the group Antibody, documents include at least one of the terms: “sarilumab”, “regeneron 3048”, “regn 3048”, “regn3048”, “regn-3048”, “regeneron 3051” “regn 3051”, “regn3051”, “regn-3051”, “sab-301”, “sab 301”, “sab301” or “siltuximab”. Finally, the group Vaccine includes documents with at least one of the following terms: “gls5300”, “gls-5300”, “gls 5300”, “chadox1 mers”, “chadox1-mers”, “mva-mers-s”, “mvamers-s”, “mrna 1273” or “mrna-1273”. All these terms were searched in the abstract, document title and database and keywords.

For the analysis of institutions, we have considered the data on “author’s affiliations - disambiguated” of documents classified as WHO’s drugs or Other drugs, Antibody and Vaccine. With the help of a script developed in R language, affiliations were checked and duplications in a single document were deleted. Thus, based on the list of institutions without duplications, it was possible to rank institutions and countries according to the frequency of documents in each group.

In order to verify whether the most prolific institutions and countries among documents on coronavirus are also the most prolific in terms of drug or therapy development against COVID-19, on 23 July 2020, we retrieved data from Cortellis, a private database owned by Clarivate Analytics. It catalogues different types of information, manually curated, on basic research and clinical development in life sciences, including technological agreements in progress stablished by two organisations in order to develop a new drug or therapy in a single platform. So, considering registers assigned as agreement, we have searched the term “coronavirus infection”, we have found 2,226 agreements, which encompass 186 institutions, 766 drugs and 879 patents. Data on the 2,226 agreements were downloaded in a Microsoft Excel format and all agreements dealing with drugs and therapies were identified acorts following.

Agreements classified as WHO’s drugs were identified by having the following terms: hydroxych, remdesivir, lopinavir, ritonavir, “interferon beta”, “beta interferon”, interferonbeta and betainterferon. As for agreements on Other drugs, we used the words darunavir, cobicistat, chloroquine, danoprevir, diammonium, glycyrrhizinate, favipiravir, leronlimab and oseltamivir. As for agreements classified as antibody and vaccine were identified, respectively, by containing at least one of the terms (a) sarilumab, “regeneron 3048”, “regn 3048”, regn3048, regn-3048, “regeneron 3051”, “regn 3051”, regn3051, regn-3051, sab-301, “sab 301”, sab301 and siltuximab and (b) gls5300, gls-5300, “gls 5300”, chadox1, mva, “mrna 1273” and mrna-1273. The affiliations of each group of agreement were identified and the frequency of institutions and countries was then calculated.

## RESULTS

The sum of the documents classified in all four studied groups (Alphacoronavirus, Betacoronavirus, Gammacoronavirus and Coronavirus) with no duplication is 31,815. This amount is distributed in the following typologies: 68% article or article in press, 10% article review, 7% letter, 5% editorial, 4% note, 3% conference paper or review, 1% book, 1% short survey and 1% data paper, erratum or retracted. As we are interested in investigating the new scientific knowledge on coronavirus, we decided to include all these documents, even books that are being produced more and more in a very short time, which greatly reduces the time gap between the process of publishing a scientific journal and a book.

In the following sections, we present the results regarding (a) general data, including the time trends of documents on each group of coronaviruses and the number of documents of Coronavirus group according to the type of research application and (b) a subset of data of Coronavirus groups related with drugs and other therapies as well as their leading institutions and countries in both scientific documents and technological agreements.

*Time trends of the four groups of documents on coronavirus* - The number of documents on coronavirus classified under the four main groups by decades is shown in Fig. 1. As can be seen, the number of documents found in the 1940s and 1950s is residual, either because of the database’s low journal coverage in these decades or because research on coronavirus was still incipient at that time.

In the 1940s, we found the first document on coronavirus registered in Scopus: a paper entitled “Demonstration of an interference phenomenon associated with infectious bronchitis virus (IBV) of chickens”, that is, a clear example of document related to one of the main MeSH terms related to Gammacoronavirus. As for the following decade, we found 18 documents in the Betacoronavirus group, being none of them related to human coronavirus but to hepatitis virus either in mouse or mice, which is in accordance to MeSH terms indicated to this family.

From the 1960s to the 2000s and the year 2020, there was a strong growth trend in the number of documents related to most groups, especially the Coronavirus group (gray bar). Documents of this group increased from 89 to 9,179, that is, a growth rate of 102.1 in the period. Considering the total of documents indexed at Scopus database (data not shown), these totals represent 0.00% and 0.44%, respectively, which signals a real increase in this thematic when compared to the whole database.

The total of documents related to the three coronavirus genera also indicates growth over the periods, except for the year 2020 in the groups of Alphacoronavirus (blue bar) and Gamacoronavirus (black bar), being the latter not associated with humans. The total number of documents in these two groups increased, respectively, from 9 to 1,492 (a growth rate of 164.8) and 56 to 1,052 (a growth rate of 17.8) from 1960s to 2010s. Considering the whole set of coronavirus documents (with no duplication) published in the 2010s (n = 8.210), the share of documents on Alphacoronavirus and Gamacoronavirus are 18.17% and 12.81% respectively.

As for Betacoronavirus (orange bar), we observe an intense growth in the whole period: from 122 in the 1960s to 5,522 in the year 2020 (a growth rate of 44.3). These totals correspond to 0.38% and 17.35 % of the whole set of coronavirus documents (with no duplication) published in the respective decades. It is noteworthy that, as observed for the coronavirus group (gray bar), documents on Betacoronavirus had a notable increase over the period, which seems to take place in two phases: from 1960s to 1990s and from 2000s to the year 2020. In this second phase, we observe a greater acceleration in the growth rate. The inclusion of new titles in the Scopus database throughout the 2000s may partially explain it while the outbreak of SARS in Asia during 2020, which reached a total of 33 countries in five continents,^33^ has boosted research on coronavirus, especially on Betacoronavirus that is associated with SARS, and so led to the notable increase in publications observed between the 1990s and 2000s.

*Types of research application among documents of coronavirus group* - As the Coronoravirus group (n. 28,013) displays the largest number of documents, we decided to investigate some general trends to better characterise it. In order to get a first insight about the relative frequency of documents on coronavirus related to treatment, where WHO’s drugs are included, in this section we present the distribution of these documents according to the type of research application they are related with, that is (a) diagnosis, (b) treatment or (c) vaccine, a classification that is supported by Tarik Jasarevic.^34^

**Fig. 2:**
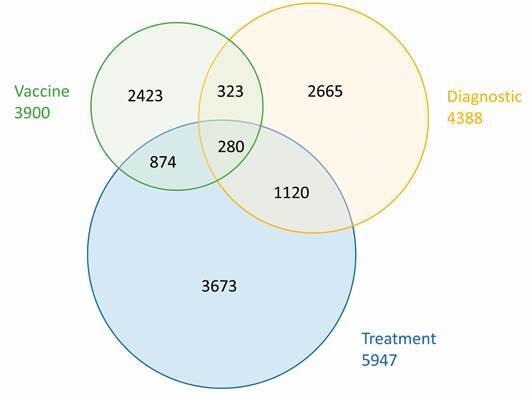
Venn diagram to the three types of research application among documents on coronavirus published in the complete period. Source: Scopus.

The analysis was carried out considering a Venn diagram (Fig. 2), which makes it possible to identify the intersections between the three types of application. We found that, over the period of analysis, the scientific community on coronavirus has been dedicating more efforts to research related to treatment (n. 5,947). The documents in this group, in general, include controlled studies with antiviral agents or with some other drugs associated with the reduction or combat of the symptoms related to the disease.

Documents related to diagnosis (n. 4,388) and vaccine (n. 3,900) are less frequent. The first group includes publications that deal with diagnostic imaging techniques, techniques for the detection of the virus and epidemiological data⁠, while the latter includes publications related to techniques and essential content for the development of a vaccine, such as the viral genome, the viral envelope proteins, neutralising antibodies, virus replication etc.

Regarding the shared zones of the diagram, documents related to treatment display the largest intersection areas, they are: with diagnosis (n. 1,400 documents) and with vaccine (n. 1,154 documents). This finding reinforces the prominent role of this type of research application (treatment) within the set of documents on coronavirus. It is also relevant to highlight the 280 documents that were identified in the intersection area that includes treatment, diagnosis and vaccine. Out of 280 documents, 130 are review articles, while the remaining are research articles focused in a more general understanding of the disease or the virus.

**Fig. 3:**
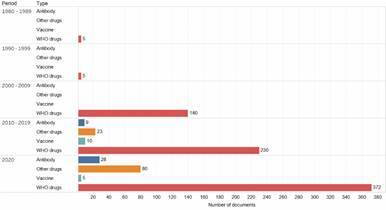
number of documents on coronavirus main groups related to drugs and other therapies by decade. Source: Scopus.

*Drugs and other therapies among documents of coronavirus group* - In this section, we identify and compare the number of documents within the Coronavirus group (n. 28,013) that are related to two groups of Drugs (WHO drug-related documents and Other Drugs) and to a group of Antibody, both with the potential for use in the treatment of COVID-19 patients. We also identify the documents within the Coronavirus group that are related to a group of Vaccines, which makes a counterpoint to the previous groups (Drugs and Antibody), since it is generally a preventive method for avoid spreading the disease. Fig. 3 presents the number of documents on coronavirus by decade according to the group of drugs or to the other therapies (antibody and vaccine) they are related with. As a first observation, we draw attention to the low number of documents in the four groups, which adds up to 907 from the 1980s to the year 2020, that is, a share of 3.3% of the total documents on Coronavirus group published in this period.

As can be noted, WHO drug-related documents (red bar), that is, those indicated in the WHO Solidarity Project, predominate largely among the documents on coronavirus in all periods. Documents included in WHO drugs sum 752, that is, 82.9% of the total number of documents on coronavirus included in the four groups of drugs or other therapies (vaccines and antibodies).

In the 2010s and in the year 2020, it is noted an increase in the number of documents on coronavirus related to Other drugs (orange bar) and Antibody (dark blue). We believe that such increase is due to the outbreak of MERS-CoV, which emerged in Saudi Arabia in 2012. The research driven by this outbreak gained more strength in late 2019, when the SARS-CoV2 pandemic started. However, we did not observe a similar trend among documents related to Vaccines (light blue) within the documents of coronavirus group. The low and reducing number of these documents in the year 2020 may be a consequence of the complex process that the development of a vaccine represents, characterised by its time duration and confidentiality, which often reduces the diffusion of the publication.

For a better understanding of the nature of the two groups of drugs and other therapies (antibody and vaccine), Table II presents the name of the compounds and their respective number of documents in each group. Within the group WHO’s drugs, documents referring to a drug named Interferon beta are the majority (n. 331), while those related to Hydroxychloroquine sulfate, which was widely propagated by some global representatives^35^ as a potential treatment for COVID-19, are the least frequent (n. 13). As for this drug, it is well-known that some papers on Hydroxychloroquine sulfate with great repercussion in society were retracted in 2020 and bring concerns about the safety of this drug,^36^ what reinforces that there is not enough scientific evidence of this drug in curing or combat COVID-19. Coincidentally or not months later, this drug was the first drug to be withdrawn from WHO’s program.

**TABLE II:** Number of scientific documents on coronavirus related to the drugs recommended by WHO’s Solidarity Program or related to drugs and other therapies listed in the Clarivate Report, 1940 to 2020

Drugs / Sources	Drugs from WHO		Drugs from Clarivate	
	Name	Nº	Name	Nº
Drugs / other drugs	Hydroxychloroquine sulfate	13	Chloroquine phospate	5
	Interferon beta	331	Danoprevir	5
	Lopinavir/ritonavir	325	Darunavir/cobicistat	16
	Remdesivir	257	Diammonium glycyrrhizinate	1
			Favipiravir	88
			Leronlimab	2
			Oseltamivir phosphate	1
Antibody			REGN 3048	4
			REGN 3051	3
			SAB-301	6
			Sarilumab	24
			Siltuximab	9
Vaccine			ChAdOx1 MERS	3
			GLS 5300	5
			mRNA 1273	4
			MVA-MERS-S	3

Source: Scopus and Cortellis.

Regarding the group Other drugs, we found documents on coronavirus related to seven drugs, among them Favipiravir, a nucleoside analog already used in the treatment of influenza A and B, appears with the largest number of documents in the period (n. 88). In the group Antibody, we identified documents on coronavirus related to five drugs, being Sarilumab, a human monoclonal antibody already used to treat rheumatoid arthritis, the one with the largest number of documents (n. 24). Finally, in the group Vaccine, the largest number of documents is related to the GLS 5300 vaccine (n. 5), a vaccine that uses plasmid DNA to express the MERS-CoV spike glycoprotein.

*Drugs and other therapies among coronavirus documents and agreements: the leading institutions and countries* - In this final section, we analyse the institutions and countries that lead the research in the four groups of drugs and other therapies according to the number of documents on Coronavirus group (collected from Scopus database) as well as the agreements to develop therapies against the coronavirus (collected from the Cortellis database).

In Table III, we first present a general analysis, where the number of documents and agreements on coronavirus are related to the number of institutions and countries within the four main groups of drugs and other therapies.

**TABLE III:** Number of documents on coronavirus and active agreements on coronavirus infection related to the drugs recommended by WHO’s Solidarity Program and related to drugs and other therapies listed in the Clarivate Report according to their respective number of institutions and countries, 1940 to 2020

Variable / Sources	Documents from Scopus				Agreements from Cortellis#			
	WHO drugs	Other drugs	Antibody	Vaccine	WHO drugs	Other drugs	Antibody	Vaccine
Nº Documents / Agreements								
With 1 institution address	270	34	12	2				
With 2 institution addresses	216	35	8	3	39	47	15	30
With 3-6 institution addresses	266	35	17	9				
Nº Institutions	1,310	260	152	38	52	62	15	33
Nº Countries	67	33	26	11	13	19	6	12

Source: Scopus and Cortellis. *#*: include technological agreements in progress established by two organisations to develop a new drug or therapy in a single platform.

In Scopus documents, we observed that documents in WHO drugs and Other drugs are distributed almost equally among the three categories of number of institutions. Documents with two or more institutions sum 482 and 70, which represent 64.1% and 67.3% in both groups, respectively. As for documents in Antibody and Vaccine, we note a quite different trend, especially the latter, with 80% of the documents signed by two or more institutions. Although documents of the latter groups tend to show a higher level of collaboration, they embrace a lower level of diversity and number of institutions and countries, when compared to the two prior groups.

As for the agreements on coronavirus, that is the Cortellis dataset, we did not observe any variation in terms of number of institutions, since all agreements refer to the participation of two institutions only, in other words, data refer to bilateral agreements. Also, we noted a lower level of institutional and country diversity, especially among agreements included in Antibody and Vaccine groups.

When compared to Scopus dataset, we observed a different trend in terms of the share of each group: the largest number of institutions within the agreements is related to Others drugs rather than to WHO’s drugs while the lowest number of institutions within agreements is related to Antibody and not to Vaccine. Although the number of institutions in agreements may be affected by its bilateral nature, we can consider that these findings indicate different institutional capabilities and interest between the two sets of data: institutions that sign documents on coronavirus retrieved from Scopus are more interested in (basic) research related to WHO’s drugs, while those that sign agreements catalogued at Cortellis are more interest in the development of other drugs. A better comprehension of the reasons associated to these differences is not the focus of the present study.

To better understand whether institutions are involved in the research on coronavirus and in the development of a treatment to prevent or to seek the cure for the virus, we investigated the top leading institutions in terms of number of documents and agreements according to the groups of drugs and therapies. These results are exposed in Table IV, where institutions that appeared more than once are highlighted in light blue (for two institutions) or in dark blue (for three institutions).

**TABLE IV:** Top ranked institutions affiliated to documents on coronavirus and to agreements on coronavirus infection related to the drugs recommended by WHO’s Solidarity Program and related to drugs and other therapies listed in the Clarivate Report, 1940 to 2020

Drugs/Sources	Documents from Scopus	Nº	Agreements from Cortellis^*#*^	Nº
WHO drugs	University of Hong Kong, Hong Kong	47	Gilead Sciences Inc, USA	13
	University of North Carolina, USA	24	Abbott Laboratories,USA	4
	Vanderbilt University, USA	19	AbbVie Inc, USA	3
	University of Pennsylvania, USA	13	Bayer Schering Pharma AG, Germany	3
	Harvard Medical School, USA	12	Synairgen plc, UK	2
	University of Texas, USA	12	Accord Healthcare Inc, USA	2
	University of Virginia, USA	12	Nat Inst of Allergy and Inf Diseases, USA	2
	University of Iowa, USA	11	Novartis AG, Switzerland	2
	Cleveland Clinic Foundation, USA	10	Triangle Pharmaceuticals Inc, USA	2
			University of Oxford, UK	2
Other drugs	University of Hong Kong, USA	6	CytoDyn Inc, USA	15
	Karolinska Institutet, Sweden	4	Drexel University College of Medicine, USA	3
	Peking University, China	4	FUJIFILM Toyama Chemical Co Ltd., Japan	3
	Nat Taiwan Univ Hospital, Taiwan	3	InterMune Inc, USA	3
	University of Virginia, USA	3	Progenics Pharmaceuticals Inc, USA	3
	Academic Medical Center, USA	2	Ajinomoto Althea Inc, USA	2
	Aix-Marseille Universit, France	2	Ascletis Pharma Inc, China	2
	Al-Faisal University, Saudi Arabia	2	FUJIFILM Holdings Corp., Japan	2
	Emory University, USA	2	Janssen Diagnostics BVBA, Belgium	2
	Fudan University, China	2	Johnson & Johnson, USA	2
			MediVector Inc, USA	2
			Nat Inst of Allergy and Inf Diseases, USA	2
			Roche Holding AG, Switzerland	2
			Russian Direct Investment Fund, Russia	2
			Toyama Chemical Co Ltd, Japan	2
Antibody	Columbia University, USA	4	Regeneron Pharmaceuticals Inc, USA	8
	Istituto Nazionale Tumori, Italy	3	EUSA Pharma, UK	4
	Harvard Medical School, USA	3	US Dep of Health and Human Services, USA	4
	Nat Inst of Allergy and Inf Diseases, USA	3	SAB Biotherapeutics Inc, USA	2
	Yale University School of Medicine, USA	3	Sanofi AS, France	2
	Macrogenics Inc., USA	2		
	Sapienza University of Rome, Italy	2		
	Johns Hopkins Univ School of Medicine, USA	2		
	National Institutes of Health, USA	2		
	Regeneron Pharmaceuticals, USA	2		
Vaccine	German Centre For Infection Research, German	4	ModeRNA Therapeutics, USA	9
	Philipps University of Marburg, German	4	AstraZeneca plc, UK	6
	University of Munich, German	3	The Jenner Institute, UK	6
	University of Oxford, UK	3	University of Oxford, UK	6
			Coalition for Epidemic Prep. Innov, Norway	4
			US Dep of Health and Human Services, USA	2

Sources: Scopus and Cortellis. *#*: include technological agreements in progress established by two organisations to develop a new drug or therapy in a single platform.

Among Scopus documents, we observed the predominance of institutions from the education sector (22 out of 30 institutions) and the presence of a few number of research institutes and corporations. In the two groups related to drugs (WHO drugs and Other drugs), the University of Hong Kong, HK, appears with the largest number of documents. Other universities also stand out in these groups, but are not listed in the other groups, as the University of Virginia, USA. Among the institutions that are listed in more than one group, apart those mentioned before, we found Harvard Medical School, USA, authoring documents on WHO drugs and Antibody.

In the group Antibody, the leadership is with Columbia University, USA. This group presents the largest variety of research institutes and corporations, such as the Istituto Nazionale Tumori, Italy, National Institute of Allergy and Infectious Diseases, USA, Macrogenics Inc. USA, National Institutes of Health, USA and Regeneron Pharmaceuticals, USA. While the group Vaccine shows three German institutions, the German Centre for Infection Research, the Philipps University of Marburg and the University of Munich and also the University of Oxford, UK. None of the institutions in this group is listed in the others.

A comparison with the top leading institutions in terms of agreements on coronavirus pointed to a completely different profile, with a predominance of private companies (29 out of 33 institutions) established in European countries and the USA, mainly. We also observed the presence of a few numbers of universities and research institutes, of which we stand out: (a) The National Institute of Allergy and Infectious Diseases in USA included in the groups WHO’s drugs and Other Drugs, (b) the University of Oxford in United Kingdom enclosed in the groups WHO’s drugs and Vaccine and (c) the US Department of Health and Human Services in USA in the groups Antibody and Vaccine. It is worth highlighting that the University of Oxford was the only institution that appeared in the agreements and in the documents in the same group of Drugs/Sources, which demonstrates their ability to develop basic and applied research with the same object of research, that resulted in documents production and in the development of drugs and other therapies, such as vaccines.

Among the private companies, we highlight the ones with greatest number of agreements: (a) in the group WHO’s drugs, the Gilead Sciences Inc, USA,^37^ that is investigating the drug Remdesevir, (b) in the group Other Drugs, the CytoDyn Inc, USA,^38^ that is carrying on research on Leronlimab which is in phase 3 clinical trial, (c) in the group Vaccine, the ModeRNA Therapeutics, USA,^39^ that is developing the mRNA-1273 vaccine, which is also in phase 3 clinical trial and (d) in the group Antibody, the Regeneron Pharmaceuticals Inc, USA, is investigating the potential of the compounds REGN 3048 and Sarilumab.

**Fig. 4:**
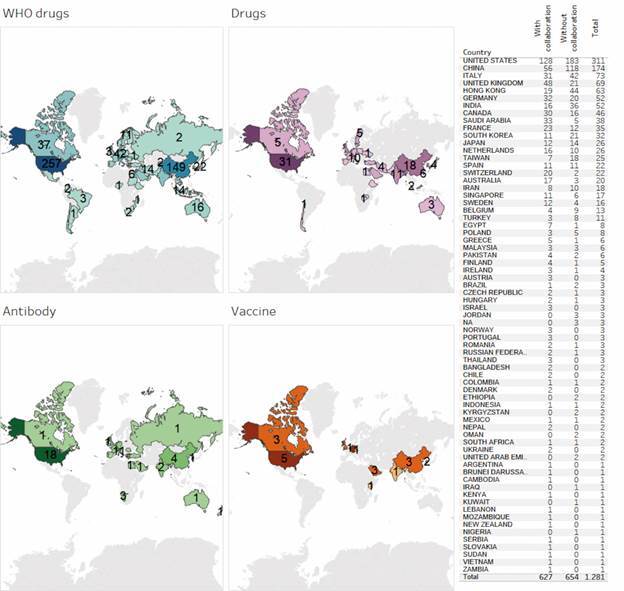
documents on coronavirus per country according to the four main groups of treatment. Source: Scopus.

**Fig. 5:**
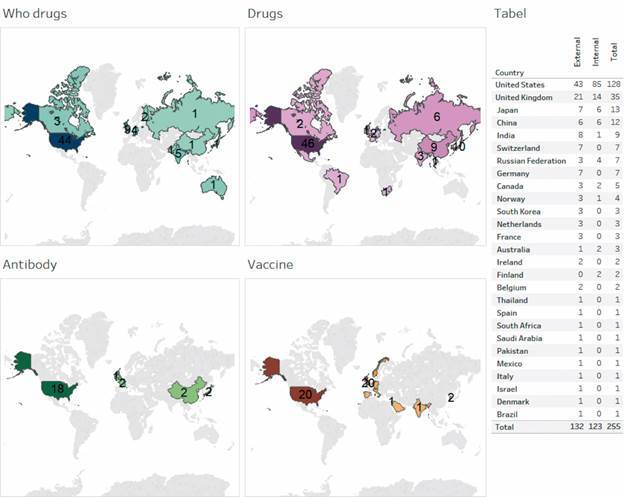
agreements on coronavirus infection per country according to the four main groups of therapy. July, 2020. Source: Cortellis.

As a final analysis, we investigated the distribution of documents and agreements on coronavirus according to the country, as shown in Figs 4-5. The colour intensity in both maps indicates the quantity of documents or agreements in each group of drugs or therapies signed by the respective country. To better visualise the contribution of each country, the maps present an attached table, which contains the total number of documents (Fig. 4) or agreements (Fig. 5) by country as well as the number of documents or agreements in collaboration (internal collaboration, that is, between institutions in the same country, and external collaboration, that is, between institutions from different countries).

Regarding the affiliated country of documents of Coronavirus group (Fig. 4), we found that the United States and China are the most prominent, with respectively 311 and 174 documents published in the whole period. The majority of these documents (67.8% for China and 58.8% for the United States) were developed without external collaboration, which indicates a high level of research independence of both countries. We emphasise that the collaboration between countries is dynamic and may change depending on the situation of the pandemic^40^ and a demonstration of this is a more intense collaboration between USA and China during the covid-19 pandemic.^41^ As for the different groups of drugs and therapies, we also observed a central role of the United States and China and these two countries also appear as leaders in production of documents on this theme in other studies.^41^ ^,^ ^42^ Besides, the maps clearly show that the research on WHO’s drugs is the one that mostly drives the attention and efforts of the scientific community, including documents signed by authors from all continents. On the other side, research on coronavirus vaccine does attract or involve a few numbers of countries from restricted regions of the world.

Considering the agreements (Fig. 5), the United States once again stands out, being the country with the largest number and share of agreements on coronavirus (almost 50%), while the United Kingdom (and not China) appeared in the second position (with almost 14%). Among the four groups of drugs and therapies, the United States leadership is unbeatable, while European countries show a lower level of involvement. It is noteworthy that none of the groups contains agreements authored by countries from all continents, as we observed in Fig. 4 for group WHO’s drugs.

Comparing the groups in Figs 4-5, we noted that some countries are found in both maps, but others are replaced or simply disappeared, as is the case of Latin American and African countries that are included in the map of WHO’s drugs in Fig. 4 but not in Fig. 5. Of course, such a different profile may be a consequence of the fact that agreements are signed by two countries only. Nevertheless, it may indicate a gap in the institutional capability between countries: in one side, most countries do have technical and human capabilities to develop basic or clinical research on coronavirus (estimated by the number of scientific documents) and in another side fewer countries display the needed capability to develop new drugs or therapies to treat or to prevent the coronavirus (estimated by agreements).

## DISCUSSION

The present study aims to investigate whether the drugs indicated by WHO’s Solidarity Project was designed upon a scientific basis, that is, whether it reflects the existing efforts of the global scientific community related with coronavirus both the level of the production of new scientific knowledge and the institutional capabilities, estimated by the most prolific institutions among documents on coronavirus and their relationship with the most prolific in the development of therapies against COVID-19. In view of these two aspects, we aimed to discuss to what extent the choice of drugs included in WHO’s Solidarity Project was based on the existing scientific knowledge and institutional capabilities. Such an approach, as far as our knowledge, is unique if we consider the lack of similar papers about research on coronavirus in world literature. Besides this characteristic, the present study does develop an innovative strategy search based in MESH terms, which made it possible to retrieve 31,815 documents on coronavirus (with no duplication). Such volume of documents is indeed higher when compared to other similar papers, in which the number of analysed documents was around 18,000.^43^ ^,^ ^44^ Hence, considering these two characteristics, we do believe in the originality, robustness and reliability of the data presented in our paper.

Among the general findings, we observed a continuous growth over the decades in three out of the four studied groups of documents, especially the one named as Coronavirus (n. 28,013). In the year 2020, we observed a big drop in the number of documents in both Alphacoronavirus and Gammacoronavirus groups, while the number of documents on Betacoronavirus increased remarkably. This is an indication of the great effort of the global scientific community to find a quickly response to the human coronavirus that is responsible for the 2020 epidemic. It is possible that part of the researchers involved with research on Alpha and Gammacoronavirus has migrated and started developing research on Betacoronavirus and so promoted such increase in the documents. A detailed analysis on authorship could confirm this assumption, but it was not the focus of the present study.

As the Coronavirus group encompasses documents of other groups, we considered it as our main source to carry on the following analyses, including the analysis related to the type of research application. With respect to this analysis, we found that the majority of documents are associated with treatment, that correspond to 22.2% of publications included in the Coronavirus group. Contrarily Lou et al.^45^ found that publications on coronavirus associated to diagnosis and treatment correspond to 10.4%, while those associated to epidemiology are the most frequent, that is 37.2%. Nevertheless, such discrepancy may be a consequence of the low number of publications on coronavirus analysed by these authors (n. 183), which made it possible a manual categorisation of the publications into different research focuses.

In a second part of our study, that is its core part, we presented findings on documents and agreements of Coronavirus group related with drugs and other therapies, specifically antibody and vaccine. It is worthy mention that the inclusion of documents related to other drugs and therapies appears as comparative dataset that allowed a better contextualisation of the relative presence and weight of documents and agreements devoted to WHO’s drugs. As for the agreements, we have used data extracted from Cortellis database once Scopus does not have similar data. Such data was considered in this study as a proxy to measure or to estimate the capability of institutions and countries towards the development of a new drug or therapy.

Hence, we showed that documents in the group WHO’s drugs are almost five times higher than in the other groups. Although we found a very low number of documents related to Hydroxychloroquine, if we consider the performance of the set of WHO’s drugs and compare it to the set of drugs of other groups, we may assume that this is a first indication that WHO’s Solidarity Project was designed upon an existing scientific basis. Nevertheless, it does not mean that all drugs included in this project had the potential to act effectively against Betacoronavirus and so in curing COVID-19. In fact, during the development of this study, hydroxychloroquine and the combined drugs lopinavir/ritonavir were shown as inefficient in the COVID-19 clinical trials and so WHO and its partner institutions discontinued their ongoing research. Negative results are part of the scientific advance and they can be used as evidence to fight against commercial and political deceivers and, in the case of hydroxychloroquine and chloroquine, we found some papers that questioned the reliability of the positive results of these drugs in curing COVID-19.^46^ ^,^ ^47^ This and the negative results could lead to a low interest of the scientific community on developing research on this drug and, consequently, we found a low number of documents related to it.

The other two drugs are still in the agenda of global science, but recent results suggest they are as not as much promising in curing or reducing mortality by COVID-19.^43^ Despite the inconsistent results about the safety and efficacy of the elected drugs, experts have pointed to many benefits that arose with this big project led by WHO, as stated by Nahid Bhadelia, from the Boston Medical Center “You’re including many different types of subgroups and populations in different parts of the world”.^48^

According to WHO, the Solidary Project counted on the participation of a wide variety of institutions and countries and so conduct clinical trials all of the world is probably due to the already existing scientific capital and structure in such institutions. Based in this premise, we investigated the frequency of institutions and countries of the four groups of documents related to drugs and therapies and we found that documents related to WHO’s drugs are the ones with the highest involvement of worldwide scientific institutions and countries (Table III and Fig. 4). This is an indication that WHO’s Solidarity Project was designed upon an existing institutional capability. A choice of other drugs by WHO that had no or very few involvements of institutions and countries would demand a more complex logistic and training and probably it would be more costly.

Our data showed that China and USA share the leadership on coronavirus documents not only in the group of WHO’s drugs but in all other groups of drugs and therapies, being most of these documents without an international collaboration. The strategic position of China and USA as the main leaders of research on coronavirus or COVID-19 is observed in some other studies,^45^ ^,^ ^49^ which corroborates their robust and qualified human workforce as well as their competitive infrastructure. The two nations do also stand out among the top ranked countries in terms of agreements to develop therapies against the coronavirus (Fig. 5). Nevertheless, we observed a smaller number of countries signing the agreements, that is, a completely different framework when compared to documents (Fig. 4), what may indicate a gap between countries in having technical and human capabilities to develop basic and/or clinical research on coronavirus and to develop new forms or products to treat or to prevent the disease.

Finally, although our findings indicated the WHO’s choice on the repurposed drugs against COVID-19 was scientific-based in terms of number of published documents and involved institutions and countries, we cannot discard that other drugs may display better performance in both variables when compared to WHO drugs. In any case, we believe that the data shown in this study illustrates that decisions by international organisations, especially in health, may have the scientific knowledge and institutional competencies as a background and not be merely bureaucratic decisions.
